# LEM domain containing 1 (LEMD1) transcriptionally activated by SRY-related high-mobility-group box 4 (SOX4) accelerates the progression of colon cancer by upregulating phosphatidylinositol 3-kinase (PI3K)/protein kinase B (Akt) signaling pathway

**DOI:** 10.1080/21655979.2022.2047556

**Published:** 2022-03-16

**Authors:** Ding Li, Ding Wang, Haofeng Liu, Xiaohui Jiang

**Affiliations:** Department of Gastrointestinal Surgery, Affiliated Cancer Hospital of Nantong University, Nantong Cancer Hospital, Nantong City, Jiangsu Province, China

**Keywords:** Colon cancer, LEMD1, SOX4, PI3K/Akt signaling pathway

## Abstract

Colon cancer is a highly malignant tumor in the digestive system. LEM domain containing 1 (LEMD1) is supposed to be a survival marker of poor prognosis in colon cancer. We aimed to explore the role and mechanism of LEMD1 in colon cancer progression. GEPIA database analyzed LEMD1 expression in colon cancer tissues and prognosis of colon cancer patients. LEMD1 expression in tumor cells was tested by RT-qPCR and western blotting. Proliferation of colon cancer cells was estimated by CCK-8 and colony formation assays. Transwell and wound healing assays were used to appraise the cell invasion and migration. Meanwhile, tube formation assays were used to evaluate angiogenesis. The possible binding sites between SRY-related high-mobility-group box 4 (SOX4) and LEMD1 were predicted by JASPAR database. Besides, SOX4 expression in colon cancer tissues and the correlation between SOX4 and LEMD1 were examined using the GEPIA database. Luciferase reporter and ChIP assays were used to verify the interaction between SOX4 and LEMD1. The expression of proteins in PI3K/Akt signaling was evaluated by western blotting. LEMD1 was overexpressed in colon cancer tissues and cells and associated with poor prognosis. Functionally, LEMD1 deficiency impeded the proliferation, migration, invasion and angiogenesis of colon cancer cells. Additionally, SOX4 had a positive correlation with LEMD1 and could bind to LEMD1 promoter. Rescue assays validated that SOX4 elevation reversed the suppressive role of LEMD1 deletion in the development of colon cancer and the expression of p-PI3K and p-AKT. Collectively, LEMD1 induced by SOX4 drove the progression of colon cancer by activating PI3K/Akt signaling.

## Graphical abstract

**Figure:**
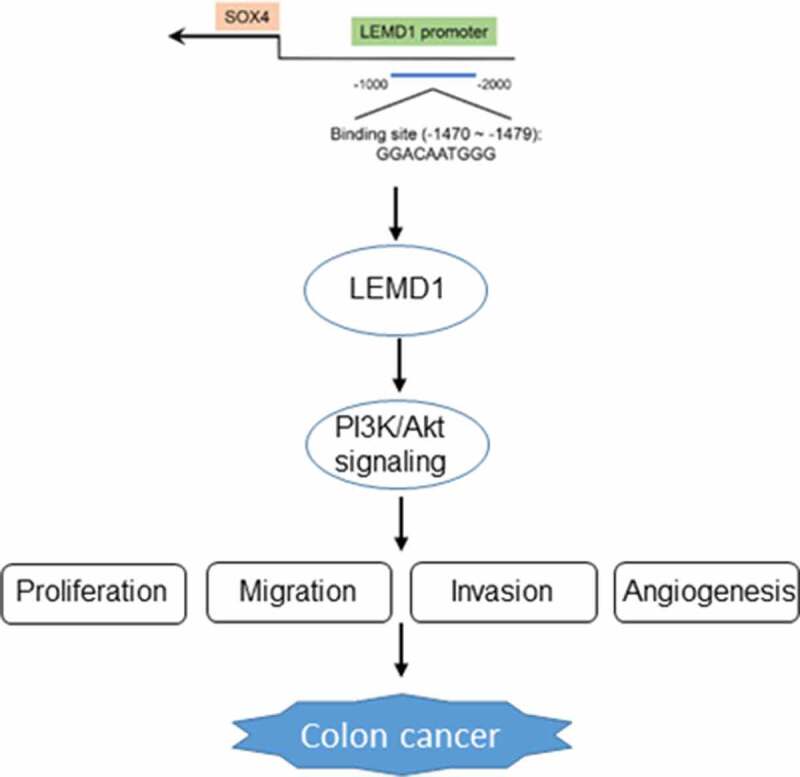

## Introduction

Colorectal cancer is deemed as the third most frequent cancer globally with a rapid rising incidence and morbidity [1]. According to statistics in 2020, approximately 1.5 million people were diagnosed with colorectal cancer and over 50, 000 deaths were caused [2]. As a matter of fact, according to the location of the lesions, colorectal cancer can be subdivided into colon cancer and rectal cancer which are commonly considered as a single disease entity [3], which also determines the differences between colon cancer and rectal cancer in terms of treatment, prognosis and metastasis [4]. As estimated, colon cancer accounts for almost 70% of colorectal cancer in 2018 [5]. The incidence and mortality rates of colon cancer are gradually increasing with 1,096,601 (6.1% of all sites) new cases and 551,569 (5.8% of all sites) deaths in 2018 globally [6]. This is due to the abnormal growth of cells in the lining or epithelium of the first and longest portion of the large intestine. It is well documented that diet, obesity, microbiome, and family history are primary factors in the etiology and pathogenesis of colon cancer [7]. In recent years, surgical and medical regimes have been improved greatly and the potential of targeted treatments has been highlighted [8]. However, colon cancer patients at advanced stages are prone to tumor metastasis and have worse survival and prognosis [9,10]. A considerable body of evidence indicates that different strategies are used to treat solid cancers including colon cancer in preclinical models [11–15]. Thus, the exploration of independent biomarkers involved in the progression of colon cancer and associated with the overall survival and prognosis of colon cancer patients is of great significance.

Cancer-testis antigens (CTAs), a family of antigens with restricted expression in testis and malignancies, are regarded as probable candidates for cancer therapy [16,17]. LEM domain containing 1 (LEMD1), a novel member of CTAs family, has also been reported to participate in various cellular processes. Take cell proliferation for an example, LEMD1 was demonstrated to exacerbate cell proliferation in gastric cancer and thyroid cancer [18,19]. In addition, high expression of LEMD1 is associated with lymph node metastasis and poor prognosis of oral squamous cell carcinoma, and contributes to tumor cell invasion and endothelial transmigration [20,21]. More importantly, LEMD1 is supposed to be a survival marker of poor prognosis in colon cancer [22]. Nevertheless, the specific functions of LEMD1 on the cell malignant phenotypes in colon cancer and the potential regulatory mechanism remain to be figured out.

SRY-related high-mobility-group box 4 (SOX4) is a common transcription factor overexpressed in diverse kinds of malignant tumors [23]. For instance, Zhang et al have supported that SOX4 functions as an oncogene in breast cancer [24]. Peng et al have elaborated that SOX4 aggravates epithelial–mesenchymal transition and stemness of gastric cancer cells [25]. The carcinogenic impacts of SOX4 on osteosarcoma have also been underlined by Chen et al. [26]. Meanwhile, the overexpression of SOX4 predicts a poor outcome of colon cancer patients [27]. Anyway, whether SOX4 binds to LEMD1 in colon cancer remains elusive.

In this study, the expression of LEMD1 in colon cancer tissues and prognosis were analyzed using the GEPIA database. Then, the functions of LEMD1 on proliferation, migration, invasion and angiogenesis of colon cancer cells were investigated. The latent regulatory mechanism of LEMD1 related to SOX4 was explored. Our findings may give rise to a new approach and theoretical basis for the treatment of colon cancer.

## Materials and methods

### Cell culture

Colon cancer cell lines (HCT116, SW480, LoVo) and human umbilical vein endothelial cells (HUVECs) were all acquired from American Type Culture Collection (ATCC, USA) while normal human intestinal epithelial cell line (HIEC) was bought from BeNa Culture Collection (Shanghai, China). The culture medium for all cells was Dulbecco’s modified Eagle’s medium (DMEM; Gibco, Grand Island, NY, USA) kept at 37°C with 5% CO_2_. 10% fetal bovine serum (FBS; GE Healthcare Life Sciences) and 1% antibiotics (Sigma-Aldrich, USA) were the addition to the medium. Every 3 months, short tandem repeat (STR) fragment analysis was used to perform cell line authentication and the mycoplasmas were tested in culture on all cell lines.

### Cell transfection

HCT116 cells were seeded into 6-well plates at a density of 2 × 10^5^ cells/well and cultured at 37°C until they reached 80% confluence. For targeted gene silencing, the specific short hairpin RNAs (shRNAs) targeting LEMD1 (sh-LEMD1#1/2) and the corresponding negative control (sh-NC) were generated by Sangon Biotech Company. Plasmids carrying SOX4 gene (Ov-SOX4) built for overexpression of SOX4 which regarded Ov-NC as the empty vector were provided by GenePharma (Shanghai, China). Cells were transfected with above plasmids employing Lipofectamine 2000 (Invitrogen, CA, USA) according to the manufacturer’s protocol. Cells were harvested 48 h later for subsequent experiments. The transfection efficiency of LEMD1 and SOX4 was examined using reverse transcription-quantitative PCR (RT-qPCR) and western blotting.

### RT-qPCR

After the preparation of total RNA from indicated cells by RNAiso reagent (TaKaRa, Dalian, China), complementary DNA (cDNA) was obtained through reverse transcription using QuantiTect Reverse Transcription kit (Qiagen GmbH) in line with the manufacturer’s guidance. Following, the amplification of cDNA templates was implemented with the application of SYBR Green PCR Master Mix (Takara, Toyobo, Japan) on the 7500 Fast Real-time PCR system (ABI, USA). The amplification process was performed as follows: 40 cycles of 95°C for 15 sec, 60°C for 15 sec and 72°C for 45 sec. LEMD1 and SOX4 expression was measured with the adoption of the 2^−ΔΔCq^ method [28] with glyceraldehyde-phosphate dehydrogenase (GAPDH) serving as an endogenous control.

### Cell Counting Kit-8 (CCK-8) assay

Colon cancer cells (5x10^3^ cells/well) were put into 96-well plates under the condition of 37°C. Following incubation for 24 h, 48 h and 72 h, 10 μl CCK-8 solution (Abcam, Cambridge, UK) was supplemented to cells using a repetitive pipette and incubated for 2 h at 37°C. Cell proliferation was measured by detection of OD values at 450 nm under a microplate reader (Bio-Rad, Hercules, CA, USA).

### Colony formation assay

A total of 1 × 10^3^ transected cells were subjected to 6-well plates. After 14 days, formed colonies were subjected to 15 min of fixation in 4% paraformaldehyde at room temperature, followed by 5 min of 0.1% crystal violet staining at room temperature. Colonies were calculated manually using an inverted microscope (Olympus Corp).

### Wound healing assay

Cells were inoculated into 6-well plates at a density of 1x10^6^/ml for incubation until 70–80% confluence. Then the scratches were created with a 200-µl pipette tip. After washing with phosphate buffer solution (PBS), the medium was then replaced with serum-free medium and cells were continued to be cultured at 37°C for 24 h. Wounds were observed at 0 h and 24 h and analyzed by an inverted microscope (Olympus Corp).

### Transwell invasion assay

To determine invasive capacity, Matrigel (Corning, NY, USA) was pre-coated into the upper chamber of the Transwell. Generally, cells seeded in serum-free medium at a concentration of 5 × 10^4^ cells per well were added to the upper transwell chambers. Meanwhile, the lower chambers were added with a medium consisting of 10% FBS. After 24 h of incubation, the invaded cells were dyed by 0.1% crystal violet for 20 min and observed under an inverted microscope (Olympus Corp).

### Tube formation assay

The angiogenic capacity of tumor cells was determined by a tube formation assay. After co-cultured with HUVECs at 37°C for 24 h, colon cancer cells were cultivated in 48-well plates precoated with Matrigel (BD Falcon, NJ, USA) at 37°C for 24 h. The ability of tube formation was observed through an inverted microscope (Olympus Corp).

### Chromatin immunoprecipitation (ChIP)

A commercially available kit (Beyotime) was utilized in ChIP assay. DNA fragments ranging from 200 to 500 bp were generated after the sonication of colon cancer cells. Following, SOX4 antibody (Santa Cruz Biotechnology, 1:1000, sc-130,633) or IgG antibody (Abcam, 1:2000, ab6728) were used to immunoprecipitated with the lysate. The purified DNA was subjected to PCR amplification.

### Luciferase reporter assay

The wild type (WT) or mutant type (MUT) sequences of LEMD1 promoter region were inserted into the pGL3 Basic vector (GenePharma, Shanghai, China) to construct the reporter plasmids of LEMD1 promoter; after that, they were co-transfected with Ov-NC and Ov-SOX4 into cells via Lipofectamine 2000 (Invitrogen, CA, USA). After 48 h, the Dual-Luciferase reporter assay system (Promega) was employed to estimate the luciferase activity.

### Western blot

Protein concentrations were ascertained by bicinchoninic acid (BCA) Protein Assay Kit (Solarbio, Beijing, China) after the preparation of total protein from cells using radioimmunoprecipitation (RIPA) lysis buffer (Beyotime, Shanghai, China). Afterward, the transfer of 50 µg proteins to polyvinylidene fluoride (PVDF) membranes (ISEQ00010, Solarbio) was conducted after proteins were segregated by 10% sodium dodecyl sulfate-polyacrylamide gel electrophoresis (SDS-PAGE). Then protein samples were probed with the following primary antibodies: anti-LEMD1 (Abcam, 1:1000, ab201206), Ki-67 (Abcam, 1:2000, ab16667), proliferating cell nuclear antigen (PCNA; Abcam, 1:1000, ab92552), matrix metallopeptidase 2 (MMP2; Abcam, 1:1000, ab92536), matrix metallopeptidase 9 (MMP9; Abcam, 1:1000, ab76003), vascular endothelial growth factor (VEGF; Proteintech, 1:1000, #66,828-1-Ig), phosphorylated-vascular endothelial growth factor receptor 2 (p-VEGFR2; Cell Signaling Technology, 1:1000, #2478 T), VEGFR2 (Cell Signaling Technology, 1:1000, #2479S), SOX4 (Santa Cruz Biotechnology, 1:1000, sc-130,633), phosphorylated-phosphatidylinositol 3-kinase PI3K (p-PI3K; Abcam, 1:1000, ab278545), PI3K (Abcam, 1:1000, ab227204), phosphorylated-protein kinase B (p-AKT; Abcam, 1:1000, ab38449), AKT (Cell Signaling Technology, 1:1000, #9272) and GAPDH (Abcam, 1:2500, ab9485) at 4°C overnight after being blotted with 5% skim milk. On the next day, the membranes were incubated with horseradish peroxidase (HRP)-linked anti-rabbit (Abcam, 1:1000, ab109489) or anti-mouse (Abcam, 1:2000, ab6789) secondary antibodies. The protein bands were visualized by the enhanced chemiluminescent (ECL) Detection Reagent (Yeasen Biotech). ImageJ software (National Institutes of Health) was used to evaluate the band density. GAPDH was used as the internal control.

### Statistical analyses

All experiments were repeated three times independently and the measurement data were exhibited as the mean ± standard deviation and GraphPad Prism software (version 8.0; GraphPad Software, Inc.) was utilized for data analysis. Differences between two groups were compared according to Student’s t-test and differences among three or more groups were compared in accordance with one-way analysis of variance (ANOVA) with Tukey’s post hoc test. Mantel-Cox test was used to determine the overall survival rate and disease-free survival rate of colon cancer patients. It was defined as statistically significant when P value is less than 0.05.

### Bioinformatics tools

LEMD1 and SOX4 expression in colon cancer tissues, the pathological stages, overall survival rate, disease-free survival rate of colon cancer patients and the correlation between SOX4 and LEMD1 in colon cancer tissues were all analyzed by GEPIA database (http://gepia.cancer-pku.cn/). The potential binding sites between SOX4 and LEMD1 promoter were predicted by JASPAR database (https://jaspar.genereg.net/).

## Results

### LEMD1 expression is markedly increased in colon cancer tissues and cell lines and concerned with poor prognosis

**Figure 1.**
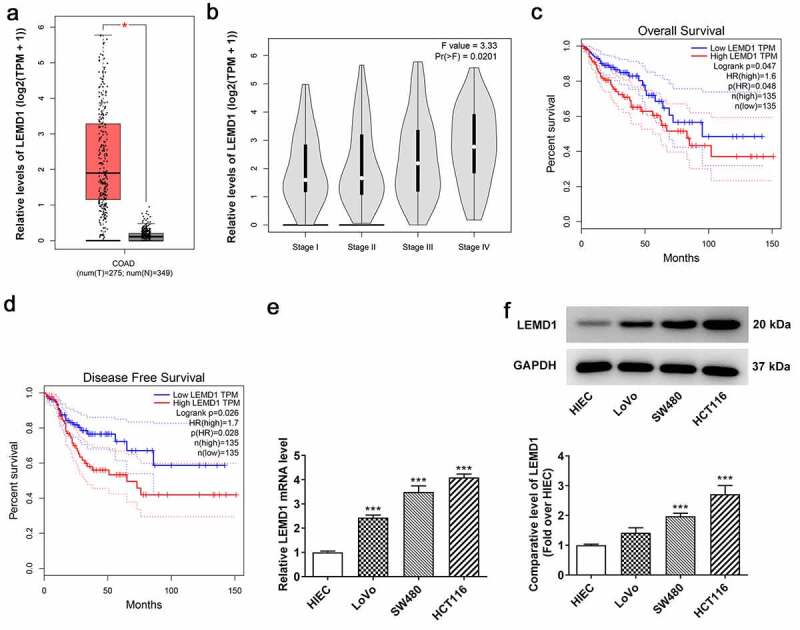
LEMD1 expression is markedly increased in colon cancer tissues and cell lines and concerned with poor prognosis. (a) Differential LEMD1 expression in tumor and adjacent tissues, the correlation of LEMD1 expression with (b) pathological stages, (c) overall survival rate and (d) disease-free survival rate in colon cancer patients were analyzed by GEPIA database. *P < 0.05. (e) RT-qPCR and (f) western blot ascertained LEMD1 expression in colon cancer cell lines and normal intestinal epithelial HIEC cells. ***P < 0.001 vs. HIEC.

It has been reported that LEMD1 is a survival marker of poor prognosis in colon cancer [22]. However, the specific functions of LEMD1 on the cell malignant phenotypes in colon cancer remain to be elucidated. To identify the role of LEMD1 in colon cancer, GEPIA database was first adopted to analyze LEMD1 expression in colon cancer tissues. As shown in Figure 1a, LEMD1 was specifically expressed in colon cancer tissues relative to normal tissues. Moreover, LEMD1 exhibited higher expression in colon cancer patients with Stage III and IV than in stage I and II (Figure 1b). It is worth mentioning that LEMD1 expressed at a notably high level was relevant to an unfavorable overall survival and disease-free survival of patients with colon cancer (Figure 1c-d). Further, the expression of LEMD1 in colon cancer cells (LoVo, SW480 and HCT116) was examined by RT-qPCR and western blot. The results implied that in comparison with human intestinal epithelial cell line HIEC, LEMD1 was prominently overexpressed in LoVo, SW480 and HCT116 cells, especially in HCT116 (Figure 1e-f). Hence, HCT116 cells were chosen for the subsequent assays. To summarize, LEMD1 was aberrantly expressed in colon cancer and might be an independent factor for overall survival of colon cancer patients.

### Insufficiency of LEMD1 hampers the proliferation, migration and invasion of HCT116 cells

**Figure 2.**
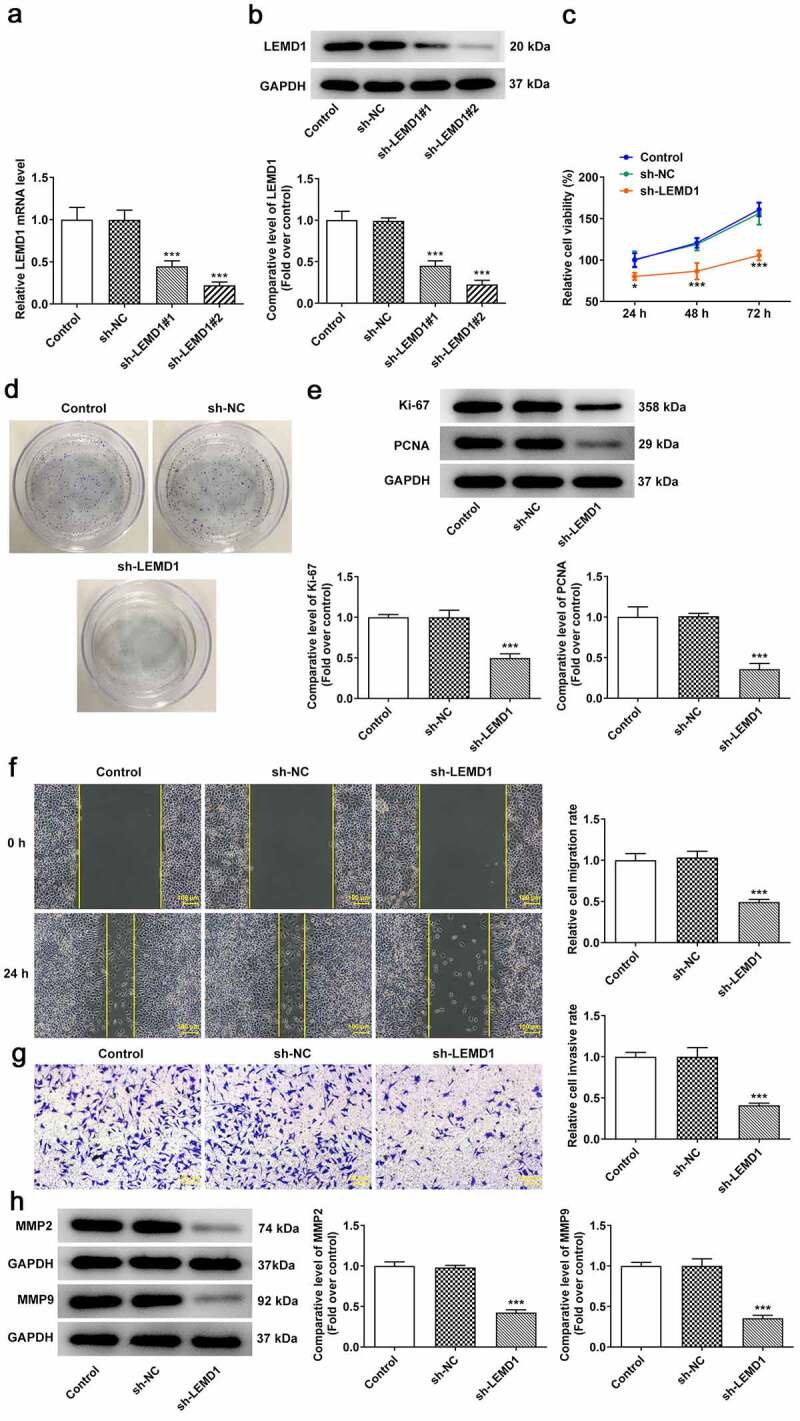
Insufficiency of LEMD1 hampers the proliferation of HCT116 cells. (a) RT-qPCR and (b) western blot analysis of the interference efficacy of LEMD1-targeted shRNAs. The proliferative ability of HCT116 cells was assessed by (c) CCK-8 and (d) colony formation assays. (e) The protein levels of Ki-67 and PCNA were determined by western blot. (f) Wound healing assay appraised cell migration. Magnification, x100. (g) Transwell assay estimated cell invasion. Magnification, x100. (h) MMP2 and MMP9 protein levels were determined by western blot. *P < 0.05, ***P < 0.001 vs. sh-NC.

To estimate the impacts of LEMD1 on the biological behaviors of colon cancer cells, LEMD1 was silenced by transfection with shRNA targeting LEMD1 in HCT116 cells. After transfection of sh-LEMD1#1/2 plasmids, LEMD1 expression was discovered to be descending conspicuously (Figure 2a-b). In the following experiments, sh-LEMD1#2 was selected for its better interference efficiency. Through CCK-8 assay, it was observed that down-regulation of LEMD1 remarkably impeded cell proliferation when compared to the sh-NC group (Figure 2c). Similarly, the colony formation ability of HCT116 cells was reduced after LEMD1 was knocked down (Figure 2d). Besides, western blot analyzed the fact that LEMD1 deficiency decreased the protein levels of proliferative markers including Ki-67 and PCNA relative to the sh-NC group (Figure 2e). Meanwhile, the experimental results from wound healing and transwell assays implied that inhibition of LEMD1 resulted in the decrease on cell migration and invasion (Figure 2f-g). To further confirm these findings, western blot was used to analyze the protein levels of invasion and migration-related factors including MMP2 and MMP9 and the results suggested that MMP2 and MMP9 expressions at protein level were both strongly reduced when LEMD1 was lowly expressed compared to the sh-NC group (Figure 2h). On the whole, LEMD1 inhibition played the suppressive role in colon cancer proliferation, migration and invasion.

### LEMD1 knockdown suppresses angiogenesis in colon cancer

**Figure 3.**
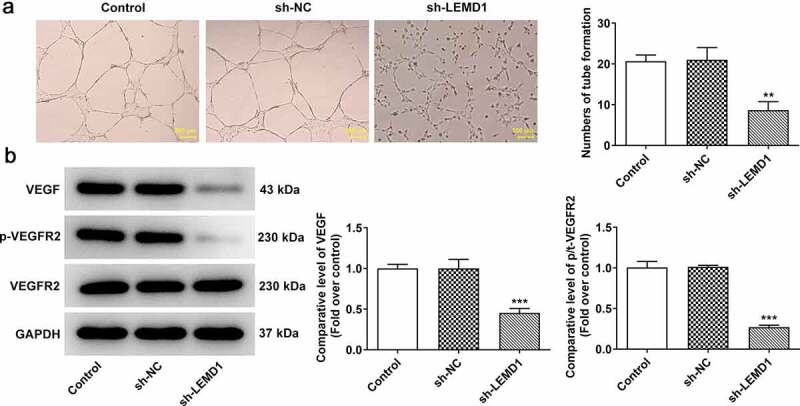
LEMD1 knockdown suppresses angiogenesis in colon cancer. (a) Tube formation assay detected angiogenesis. Magnification, x4. (b) The protein levels of VEGF and p-VEGFR2/VEGFR2 were determined by western blot. **P < 0.01, ***P < 0.001 vs. sh-NC.

Angiogenesis has been reported to play an important role in the development of tumor metastasis and inhibition of this process can significantly prevent the development and spread of tumor [29,30]. Through tube formation assay, it turned out that reduction of LEMD1 hindered tube formation of HUVECs (Figure 3a). In addition, the expressions of VEGF, a dominant regulator, and its receptor VEGFR2 and p-VEGFR2 were measured and western blot analysis indicated that shortage of LEMD1 cut down the protein levels of VEGF and p-VEGFR2/VEGFR2 (Figure 3b). In a word, silencing of LEMD1 played an anti-angiogenesis role in colon cancer.

### The up-regulation of LEMD1 in colon cancer is mediated by SOX4 transcription factor

**Figure 4.**
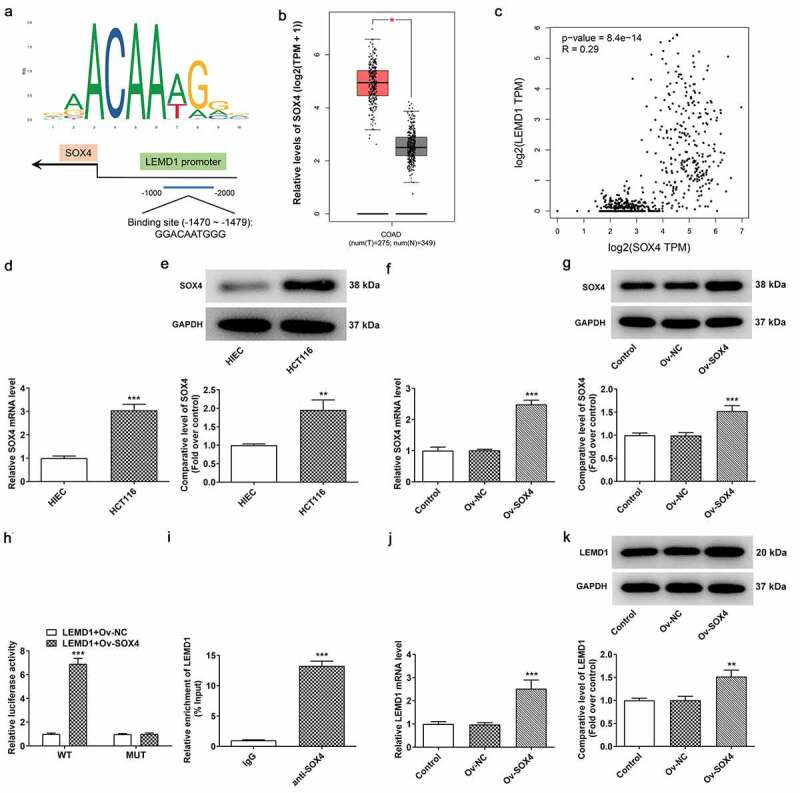
The up-regulation of LEMD1 in colon cancer is mediated by SOX4 transcription factor. (a) The potential binding sites between LEMD1 promoter and SOX4 were predicted by JASPAR database. (b) Differential SOX4 expression in tumor and adjacent tissues and (c) the correlation between LEMD1 and SOX4 were analyzed by GEPIA database. (d) RT-qPCR and (e) western blot ascertained SOX4 expression in HCT116 and HIEC cells. **P < 0.01, ***P < 0.001 vs. HIEC. (f) RT-qPCR and (g) western blot tested transfection efficiency of Ov-SOX4. ***P < 0.001 vs. Ov-NC. (h) Luciferase reporter assay verified the luciferase activity of WT and MUT LEMD1 promoter. ***P < 0.001 vs. LEMD1+ Ov-NC. (i) ChIP assay identified the accumulation of LEMD1 promoter in SOX4 antibody. ***P < 0.001 vs. IgG. (j) RT-qPCR and (k) western blot ascertained LEMD1 expression after SOX4 was overexpressed. **P < 0.01, ***P < 0.001 vs. Ov-NC.

To explore the potential mechanisms of LEMD1 on the regulation of colon cancer progression, JASPAR database was used to predict the transcription factors that could regulate LEMD1 expression. LEMD1 promoter was noticed to bind to SOX4 transcription factor, and the predicted binding sequences are exhibited in Figure 4a. Intriguingly, the high expression of SOX4 in colon cancer tissues was highlighted by GEPIA database (Figure 4b). Correlation analysis of SOX4 and LEMD1 uncovered that SOX4 had a positive correlation with LEMD1 in colon cancer (Figure 4c). Also, RT-qPCR and western blot analysis hinted that SOX4 expression was upregulated in HCT116 cells compared with HIEC cells (Figure 4d-e). Then, the overexpression efficiency of SOX4 was checked by RT-qPCR and western blot analysis and SOX4 expression was observably elevated in HCT116 cells transfected with Ov-SOX4 plasmid as comparison to the empty group (Figure 4f-g). Furthermore, luciferase reporter assay testified that SOX4 up-regulation raised the luciferase activity of LEMD1-WT instead of LEMD1-MUT (Figure 4h). ChIP assay also proved that LEMD1 promoter was enriched in SOX4 antibody (Figure 4i). Additionally, it was noticed that LEMD1 expression was increased after SOX4 was overexpressed (Figure 4j-k). All in all, SOX4 could bind to LEMD1 promoter and positively regulated LEMD1 expression in colon cancer cells.

### LEMD1 mediated by SOX4 contributes to cell proliferation, migration and invasion in colon cancer cells

**Figure 5.**
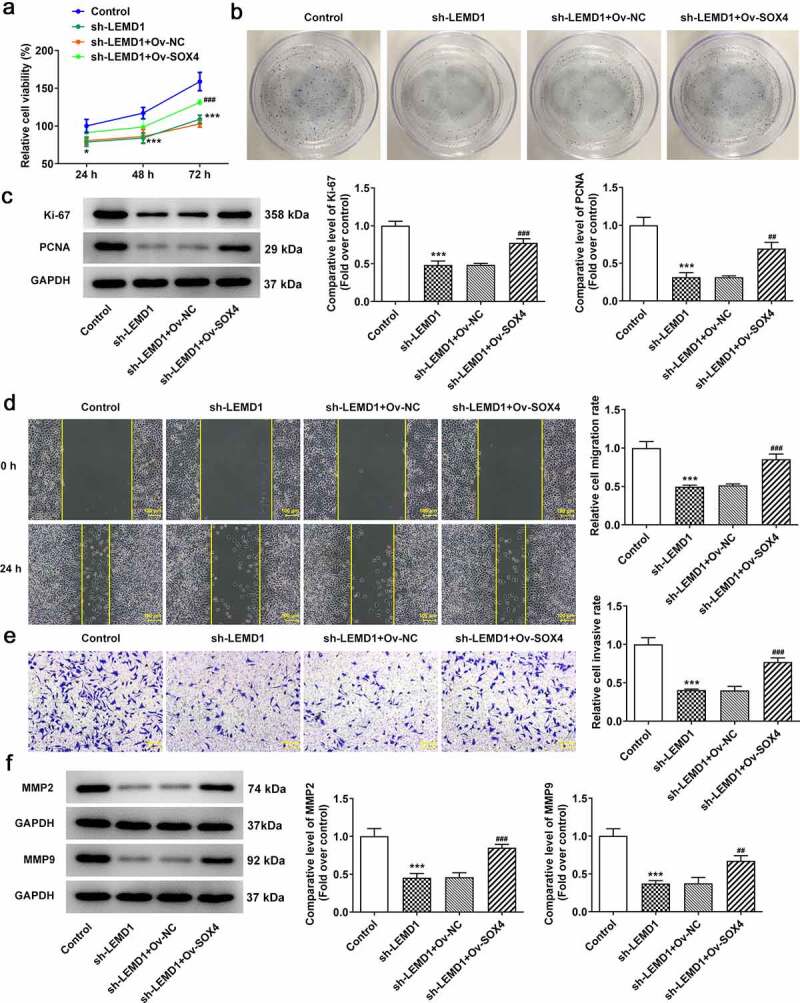
LEMD1 mediated by SOX4 contributes to cell proliferation, migration and invasion in colon cancer cells. The proliferative ability of HCT116 cells was assessed by (a) CCK-8 and (b) colony formation assays. (c) The protein levels of Ki-67 and PCNA were determined by western blot. (d) Wound healing assay appraised cell migration. Magnification, x100. (e) Transwell assay estimated cell invasion. Magnification, x100. (f) MMP2 and MMP9 protein levels were determined by western blot. *P < 0.05, ***P < 0.001 vs. control; ^##^*P* < 0.01, ^###^*P* < 0.001 vs. sh-LEMD1+ Ov-NC.

To confirm whether SOX4 mediated the biological functions of LEMD1 on colon cancer, rescue assays were conducted. CCK-8 and colony formation assays elucidated that the decreased proliferation of HCT116 cells accompanied with the reduced protein levels of Ki-67 and PCNA caused by LEMD1 depletion was restored by overexpression of SOX4 (Figure 5a-c). Likewise, up-regulation of SOX4 reversed the attenuated migratory and invasive capacities as well as declined MMP2 and MMP9 protein levels on account of LEMD1 silencing (Figure 5d-f). Taken together, LEMD1 mediated by SOX4 drove the progression of colon cancer.

### LEMD1 mediated by SOX4 induces angiogenesis in colon cancer

**Figure 6.**
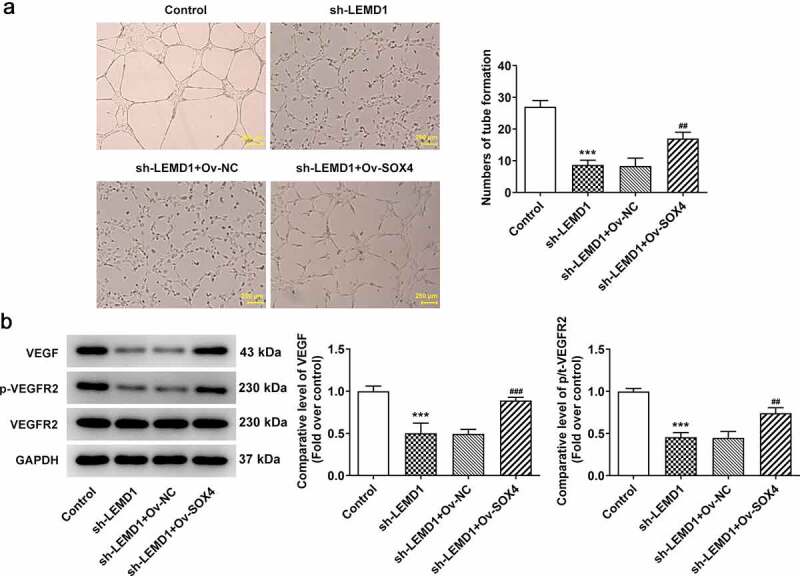
LEMD1 mediated by SOX4 induces angiogenesis in colon cancer cells. (a) Tube formation assay detected angiogenesis. Magnification, x4. (b) The protein levels of VEGF and p-VEGFR2/VEGFR2 were determined by western blot. ***P < 0.001 vs. control; ^##^*P* < 0.01, ^###^*P* < 0.001 vs. sh-LEMD1+ Ov-NC.

The subsequent experiments were performed to further investigate whether LEMD1 affected colon cancer angiogenesis was regulated by SOX4. In the same way, when SOX4 was highly expressed, the repressed tube formation of HUVECs transfected with sh-LEMD1 plasmids was facilitated again (Figure 6a). Western blot analysis also indicated that LEMD1 down-regulation led to the decrease on the protein levels of VEGF and p-VEGFR2/VEGFR2 whereas this influence was counteracted by SOX4 overexpression (Figure 6b). Briefly, LEMD1 mediated by SOX4 exacerbated angiogenesis in colon cancer.

### SOX4-stimulated LEMD1 activates the PI3K/AKT signaling in colon cancer cells

**Figure 7.**
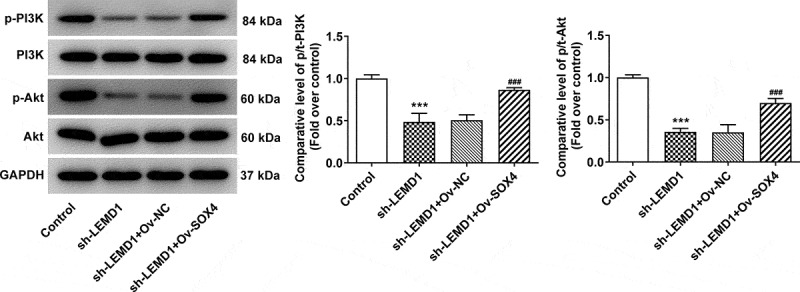
SOX4-induced LEMD1 activates the PI3K/AKT signaling in colon cancer cells. Western blot analyzed the protein levels of p-PI3K/PI3K and p-AKT/AKT in HCT116 cells. ***P < 0.001 vs. control; ^###^*P* < 0.001 vs. sh-LEMD1+ Ov-NC.

It is well documented that PI3K/AKT signaling is determined as a therapeutic target for colon cancer [31]. Consistent with this finding, western blot also analyzed that interference of LEMD1 cut down the protein levels of p-PI3K/PI3K and p-AKT/AKT and this outcome was partially offset by SOX4 up-regulation (Figure 7). Collectively, LEMD1 activated by SOX4 served as an activator of PI3K/AKT signaling.

## Discussion

Colon cancer is one of the most universal and lethal malignancies [1]. Momentous differences in phenotypic features including proliferation, migration, invasion are usually displayed during the onset and aggressiveness of colon cancer owing to tumor heterogeneity [32]. As is known to all, angiogenesis accounts for tumor growth and metastasis and has been acknowledged as a promising target in cancer therapy [33,34]. Previous studies have documented that the emergence of molecular hallmarks is an encouraging advancement for the diagnosis and prognosis of colon cancer and molecular-targeted therapy is pivotal for the treatment of colon cancer [35,36]. The role of LEMD1 has been widely investigated in a variety of human cancers, such as gastric cancer [37], thyroid cancer [19], oral squamous cell carcinoma [20,21] and all results implied that LEMD1 may act as a potent oncogene in cancers. What is more, Luo et al. have elaborated that LEMD1 involved in competing endogenous RNA mechanism contributes to the development of colorectal cancer [38]. Emerging evidence supports that LEMD1 is overexpressed and can be regarded as a survival marker of poor prognosis in colorectal cancer [22]. Consistent with these findings, LEMD1 expression was noticed to be prominently increased in colon cancer tissues and cells in the present study. Besides, LEMD1 expression differed based on pathological stages of colon cancer, as evidenced by the fact that LEMD1 exhibited higher expression in colon cancer patients with Stage III and IV than in stage I and II. Also, high expression of LEMD1 predicted a lower overall survival rate and disease-free survival rate of colon cancer patients, suggesting that LEMD1 could be used as a prognostic marker for colon cancer. In the subsequent in vitro experiments, after LEMD1 was depleted, the proliferation of colon cancer cells was observably impeded, as accompanied with the declined protein levels of proliferation markers Ki-67 and PCNA [39] and invasion- and migration-related factors MMP2 and MMP9 [40]. VEGF represents a growth factor with important pro-angiogenic activity [41]. As its receptor, VEGFR2 is also engaged in tumor angiogenesis [42]. Our experimental results also validated that interference of LEMD1 ameliorated angiogenesis and reduced VEGF and p-VEGFR2/VEGFR2 protein levels.

SOX4 is a member of the group C subfamily of SOX transcription factors [23]. In most cases, SOX4 participates in various developmental processes through gene regulation. For instance, CXCL12 is activated by SOX4 to drive the process of hepatocellular carcinoma [43]. MEM2 is transcriptionally activated by SOX4 in breast cancer [44]. Furthermore, Wu et al. have demonstrated that SOX4 binds to Cyr61 promoter in colon cancer [45]. Through our investigation, SOX4 was postulated to interact with LEMD1 promoter and the possible binding sites were predicted by JASPAR database. The strong affinity of SOX4 with LEMD1 promoter was testified by mechanism assays. Meanwhile, SOX4 and LEMD1 had a positive correlation in colon cancer tissues and cells. A growing body of evidence has substantiated that SOX4 expression is positive in various human cancers and is responsible for tumorigenesis [23,46,47]. It is noteworthy that SOX4 may serve as a prognostic factor in colon cancer [27]. A great number of reports have also claimed that SOX4 functions as a promoter in colon cancer [48,49]. In the same way, the experimental results in this study also indicated that SOX4 was distinctly overexpressed in colon cancer tissues and cells. Functional experiments also confirmed that the suppressive role of LEMD1 knockdown in the proliferation, migration, invasion and angiogenesis of colon cancer cells was reversed by elevation of SOX4.

The aberrant activation of PI3K/AKT signaling pathway composed of core components including PI3Ks and their downstream mediator AKTs are concerned with dysregulation of cell growth, proliferation, survival and angiogenesis [50–52]. At the same time, the role of PI3K/AKT signaling has been highlighted in various cancers, including colon cancer, by accumulating evidence [31,53,54]. Meanwhile, LEMD1 is an activator of PI3K/AKT signaling in gastric cancer and colorectal cancer [18,38]. Furthermore, SOX4 stimulates PI3K/AKT signaling in multiple human diseases, including acute myeloid leukemia [55], breast cancer [56], pancreatic cancer [57] and prostate cancer [58]. Similarly, it was discovered in our study that the protein levels of p-PI3K/PI3K and p-AKT/AKT were cut down by deficiency of LEMD1 whereas this outcome was restored by up-regulation of SOX4, which suggested that SOX4-mediated LEMD1 led to the activation of PI3K/AKT signaling. However, this study mainly focused on the bioinformatic analysis and in vitro experiments to explore the effects of LEMD1 in colon cancer. The lack of an analysis of clinical samples and in vivo animal experiments are limitations of the present study, which will be conducted in the future experiments to further support the conclusion.

## Conclusion

To sum up, this study uncovered the impacts of LEMD1 on the malignant properties of colon cancer cells and identified that LEMD1 transcriptionally activated by SOX4 stimulated the PI3K/AKT signaling, which for the first time revealed the mechanism by which LEMD1 facilitated the development of colon cancer.

## Data Availability

All data included in this study are available upon request through contact with the corresponding author.
